# Orelabrutinib plus reduced-dose whole brain radiotherapy in elderly primary central nervous system lymphoma: a case report

**DOI:** 10.3389/fonc.2025.1693481

**Published:** 2025-11-03

**Authors:** Qiao Wang, Hua Wang, Duozhuang Tang, Yuanyuan Wu, Siyao He, Si Tao

**Affiliations:** ^1^ Department of Oncology, The Second Affiliated Hospital, Jiangxi Medical College, Nanchang University, Nanchang, Jiangxi, China; ^2^ Jiangxi Province Key Laboratory of Precision Cell Therapy (2024SSY06241), The Second Affiliated Hospital, Jiangxi Medical College, Nanchang University, Nanchang, Jiangxi, China; ^3^ Department of Hematology, The Second Affiliated Hospital, Jiangxi Medical College, Nanchang University, Nanchang, Jiangxi, China; ^4^ Jiangxi Provincial Key Laboratory of Hematological Diseases (2024SSY06052), Department of Hematology, The Second Affiliated Hospital, Jiangxi Medical College, Nanchang University, Nanchang, Jiangxi, China

**Keywords:** PCNSL, BTKi, rd-WBRT, elderly patients, chemotherapy-ineligible, neurotoxicity

## Abstract

**Background:**

Primary central nervous system lymphoma (PCNSL) primarily affects elderly individuals, many of whom are unable to tolerate standard high-dose methotrexate (HD-MTX) chemotherapy due to frailty and comorbid conditions. There is a pressing need for alternative treatment strategies that offer reduced toxicity while maintaining therapeutic efficacy.

**Case presentation:**

In this case report, we describe three elderly patients (aged 70–78 years) with newly diagnosed, chemotherapy-ineligible PCNSL who were treated with a combination of reduced-dose whole brain radiotherapy (rd-WBRT, <30 Gy) and oral orelabrutinib (150 mg daily).

**Conclusion:**

These preliminary findings suggest that the all patients initially achieved either complete remission (CR) or partial remission (PR). Two patients maintained durable remission, whereas one patient experienced disease relapse after discontinuing orelabrutinib and switching to an alternative regimen. No significant neurotoxicity or treatment-related complications were observed. Combination of orelabrutinib and rd-WBRT may represent a safe and effective therapeutic approach for elderly patients with PCNSL who are not candidates for standard chemotherapy. Prospective clinical trials are warranted to further evaluate this approach.

## Introduction

Primary central nervous system (CNS) lymphoma (PCNSL) is a rare and aggressive form of extranodal non-Hodgkin lymphoma that arises within the brain, spinal cord, leptomeninges, or eyes, typically without systemic involvement. It accounts for approximately 4% of primary central nervous system tumors and 4–6% of all extranodal lymphomas (1). PCNSL is most commonly of the diffuse large B-cell lymphoma (DLBCL) subtype, and its incidence has been increasing, particularly among older adults, nearly half of whom are over 60 years of age at diagnosis (2–4).

Clinical outcomes in elderly patients with PCNSL are generally poor, largely due to frailty, comorbidities, and limited tolerance to intensive therapies (5). Among those receiving first-line treatment, patients under the age of 60 exhibit a median progression-free survival (PFS) of 28.4 months, with median overall survival (OS) not yet reached. In contrast, patients aged 60 and above have a median PFS of only 8 months and a median OS of 15.4 months (2).The current standard of care consists of HD-MTX–based chemotherapy, often followed by whole brain radiotherapy (WBRT) or autologous stem cell transplantation (ASCT) (1). However, HD-MTX is associated with significant toxicity in older patients, and WBRT carries a well-documented risk of long-term neurocognitive impairment, particularly at doses exceeding 30–40 Gy (6, 7). As an alternative, reduced-dose WBRT (rd-WBRT; <30 Gy) has been proposed to preserve efficacy while minimizing neurotoxicity, although its curative potential remains under investigation (8, 9).

Bruton’s tyrosine kinase (BTK) plays a pivotal role in B-cell receptor (BCR) signaling, and its aberrant activation contributes to the pathogenesis of several B-cell malignancies (10). BTK inhibitors (BTKIs) interrupt BCR signaling by inhibiting BTK, thereby suppressing downstream nuclear factor κB (NF-κB) activation, a key driver of lymphomagenesis (11). Recurrent mutations in MYD88 L265P and CD79B, frequently observed in PCNSL, promote constitutive activation of these pathways, making BTK a rational therapeutic target (12). As a result, BTK is considered an attractive therapeutic target for this disease. Orelabrutinib, a second-generation, highly selective BTKi with strong CNS penetration, has shown promise as a tolerable and potentially effective option for patients who are ineligible for standard chemotherapy (13, 14).

This case series describes three elderly patients with newly diagnosed, chemotherapy-ineligible PCNSL who received a novel treatment regimen combining rd-WBRT and orelabrutinib. Our findings indicate that all three patients achieved either complete remission (CR) or partial remission (PR). Two patients maintained durable remission, whereas one experienced disease relapse after discontinuing orelabrutinib and switching to an alternative regimen, suggesting that this regimen may represent a viable and well-tolerated option for this challenging patient population.

## Case presentations

### Case 1

A 72-year-old woman presented in January 2023 with a two-month history of dizziness, dysarthria, and gait instability. Her past medical history included cerebral infarction, congenital coronary artery–pulmonary artery fistula, and hypertension. On admission, systemic assessment revealed severe functional and cognitive impairment: Eastern Cooperative Oncology Group (ECOG) performance status was 4, and Mini-Mental State Examination (MMSE) score was 13, indicating moderate to severe cognitive dysfunction.

**Figure 1:**
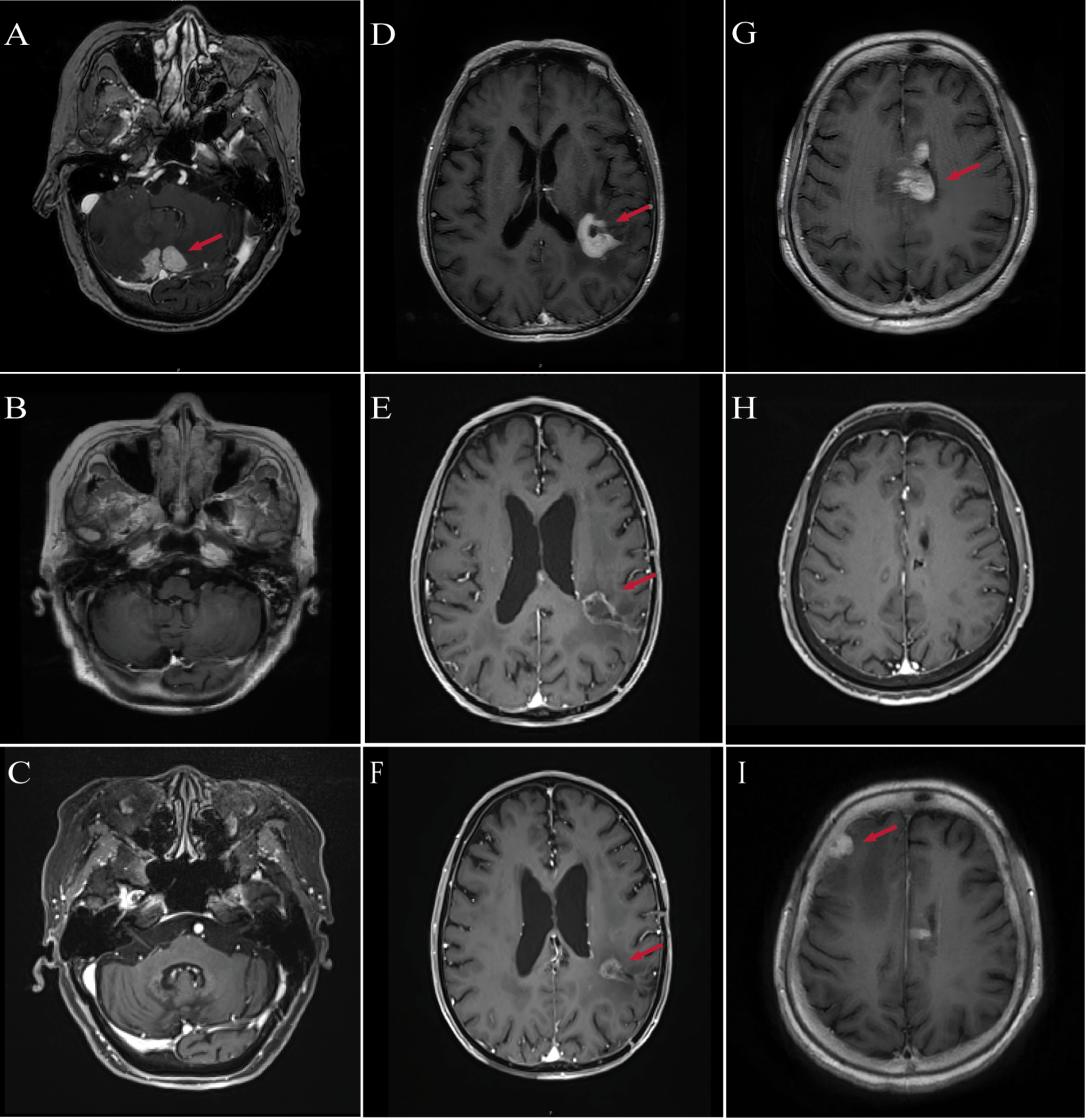
MRI imaging of patients diagnosed with PCNSL. **(A)** Patient #1: Baseline contrast-enhanced MRI shows bilateral cerebellar lesions (red arrows), consistent with PCNSL. **(B)** Patient #1: Follow-up MRI at 3 months post-treatment shows complete resolution of cerebellar lesions. **(C)** Patient #1: MRI at 29 months post-treatment confirms sustained complete remission. **(D)** Patient #2: Baseline MRI reveals enhancing lesions in the left parietal lobe, midbrain, and thalamus (red arrows). **(E)** Patient #2: MRI at 3 months post-treatment reveals a 40% reduction in tumor size compared with baseline. **(F)** Patient #2: MRI at 7 months post-treatment shows approximately 50% reduction in lesion diameter relative to baseline. **(G)** Patient #3: Baseline MRI reveals multifocal lesions involving the periventricular areas, lateral ventricles, fourth ventricular ependyma, and corpus callosum (red arrows). **(H)** Patient #3: MRI at 3 months post-treatment shows near-complete remission of previously noted lesions. **(I)** Patient #3: MRI at 10 months post-treatment reveals recurrent enhancing lesions in the right frontotemporal region and basal ganglia.

Contrast-enhanced brain MRI demonstrated multiple hyperintense lesions in the bilateral cerebellar hemispheres, leptomeninges, and cerebellar tentorium, suspicious for PCNSL (**Figure 1A**). Stereotactic brain biopsy on February 26, 2023 confirmed DLBCL, non-germinal center B-cell (non-GCB) subtype. Positron emission tomography–CT (PET/CT) and bone marrow biopsy excluded systemic involvement. The International Extranodal Lymphoma Study Group (IELSG) score was 3, indicating intermediate risk.

The patient was deemed ineligible for HD-MTX therapy, primarily due to her congenital coronary artery–pulmonary artery fistula and hypertension, which posed a high risk of cardiac decompensation during the intensive hydration required for HD-MTX. A formal cardiology consultation explicitly advised against high-volume hydration. Her general frailty and comorbidities further reinforced the contraindication for HD-MTX. She was initiated on rd-WBRT (27 Gy in 18 fractions) and daily oral orelabrutinib (150 mg). Given her history of cerebral infarction, she had been prescribed indobufen, a platelet aggregation inhibitor, which was discontinued following consultation with the neurology team regarding bleeding risks, while orelabrutinib was continued.

Follow-up MRI in March 2023 demonstrated PR, and by June 2023, CR was achieved (**Figure 1B**). Neurological function significantly improved, with ECOG performance status improved to 3 and MMSE increasing to 20, indicating mild-to-moderate cognitive impairment. In January 2024, she sustained multiple fractures from a fall, resulting in reduced mobility, but continued on orelabrutinib therapy. As of the most recent follow-up in August 2025, she remains in CR without evidence of disease recurrence (**Figure 1C**).

### Case 2

A 78-year-old man presented in February 2024 with a one-month history of progressive memory decline. His past medical history included hypertension, coronary artery disease, and bronchiectasis. On admission, ECOG performance status was 2, and MMSE 20.

Contrast-enhanced MRI revealed abnormal enhancement in the left parietal lobe, midbrain, and thalamus (**Figure 1D**). On February 28, 2024, the patient underwent neuro-navigation-guided tumor resection. Postoperatively, he developed aphasia, dysphagia, and right-sided hemiplegia. His functional status deteriorated to ECOG 4, while cognitive function remained MMSE 20. Histopathology confirmed non-GCB DLBCL. PET/CT and bone marrow biopsy excluded systemic involvement. The IELSG score was 2, indicating intermediate risk.

HD-MTX–based chemotherapy was deemed unsafe due to the patient’s advanced age, postoperative neurological deficits, and comorbidities (coronary artery disease and bronchiectasis), which increased the risk of systemic toxicity and organ decompensation. Multidisciplinary evaluation confirmed that the patient was chemotherapy-ineligible. On March 26, 2024, he was initiated on rd-WBRT (22.5 Gy in 15 fractions) and oral orelabrutinib (150 mg daily).

MRI in June 2024 demonstrated marked reduction of the intracranial lesions and decreased enhancement around the resection cavity. Clinically, neurological function improved, including memory, speech, and right-sided limb strength. Functional status recovered to ECOG 2, and MMSE improved to 22. Radiological assessment showed PR (**Figure 1E**). As of the latest follow-up in October 2024, the patient remains in PR (**Figure 1F**).

### Case 3

A 70-year-old woman presented in August 2024 with a two-month history of worsening dizziness, headache, and nausea, accompanied by lower limb weakness (grade 4) and altered mental status. Her past medical history included diabetes mellitus, hypertension, and a prior cerebral infarction. On admission, systemic assessment revealed severe functional and cognitive impairment: ECOG performance status was 4, and MMSE score was 15, indicating moderate cognitive dysfunction.

Contrast-enhanced brain MRI demonstrated multiple abnormal lesions near the bilateral lateral ventricles, fourth ventricle ependyma, and corpus callosum (**Figure 1G**). Cerebrospinal fluid analysis showed elevated protein (2+). Stereotactic brain biopsy on September 11, 2024 confirmed non-GCB DLBCL. PET/CT and bone marrow biopsy excluded systemic involvement. The IELSG score was 4, indicating high-risk disease. Postoperatively, the patient developed severe pneumonia, which further compromised her general condition.

Given her postoperative pneumonia, multiple comorbidities, and poor performance status, the patient was considered ineligible for HD-MTX–based chemotherapy. Following multidisciplinary team discussion, she received induction therapy in the Hematology Department consisting of rituximab (500 mg) combined with oral orelabrutinib (150 mg daily), achieving CR after four cycles (**Figure 1H**). On December 5, 2024, she was transferred to the Oncology Department and underwent rd-WBRT (23.5 Gy in 13 fractions) while continuing oral orelabrutinib. Follow-up MRI in March 2025 showed sustained CR, accompanied by improvement in neurological function, with ECOG performance status improving from 4 to 3 and MMSE score increasing from 15 to 18.

However, due to the high financial cost of orelabrutinib, treatment was modified on March 18, 2025 to lenalidomide (10 mg daily) plus rituximab. At the subsequent follow-up on July 1, 2025, MRI demonstrated disease progression (**Figure 1I**). Consequently, the regimen was adjusted to rituximab (500 mg) combined with oral orelabrutinib (150 mg daily) for maintenance therapy. As of the latest follow-up, her performance and cognitive status have remained stable, and repeat imaging is pending.

## Discussion

Elderly patients with PCNSL are particularly vulnerable, often facing poor prognoses and heightened sensitivity to treatment-related toxicities. Those over 70 years of age tend to show limited improvement in outcomes, likely due to factors such as reduced performance status, higher rates of comorbidities, and the use of less aggressive treatment regimens (3, 4). Methotrexate-based chemotherapy remains the standard first-line treatment for these patients. However, patients who are ineligible for standard methotrexate-based chemotherapy due to frailty or comorbidities are sometimes managed with radiotherapy, monotherapy, or supportive care (1). In a prospective study by Nelson et al., the median OS among 41 patients treated with WBRT alone was 11.6 months. Notably, the median survival was 23.1 months for the 14 patients under 60 years old, compared to just 7.6 months for the 27 patients aged 60 or older (log-rank p = 0.001) (15). In contrast, patients receiving supportive care alone had a median survival of only 3.3 months (16). While novel agents for treating PCNSL, such as BTK inhibitors, lenalidomide, pomalidomide, and nivolumab, show therapeutic potential, they are unlikely to serve as curative monotherapies (17). This highlights the urgent need for alternative treatment strategies. In this context, we report a case series of three elderly patients treated with rd-WBRT combined with the BTK inhibitor orelabrutinib. All patients achieved partial or complete remission, suggesting that this combination may represent a potentially effective and tolerable option for select patients, although its role remains investigational and requires further study.

Several BTKIs, including ibrutinib, acalabrutinib, zanubrutinib, tirabrutinib, and orelabrutinib, are approved for the treatment of hematologic malignancies. Most PCNSL cases harbor MYD88 L265P and CD79B mutations, BTKIs have shown promising efficacy in relapsed or refractory cases (12, 18, 19). More recently, studies have begun to explore the role of BTKIs in newly diagnosed PCNSL, both as monotherapy and in combination with other treatments, and recent trials have validated incorporation of BTKIs into initial therapy improves efficacy (20–22). Among these agents, orelabrutinib—a highly selective second-generation BTK inhibitor with fewer off-target effects—has garnered significant attention for its potential in treating PCNSL. A retrospective analysis of 86 PCNSL patients demonstrated a 96.2% objective response rate (ORR) with BTKIs combination therapy, compared to 71.4% with traditional chemotherapy and 71.4% with radiotherapy. The median OS was also significantly better in the BTKi group, with a median OS not reached versus 47.8 months in the chemotherapy group (P = 0.038) (23). Another analysis of orelabrutinib-based regimens in newly diagnosed or relapsed/refractory PCNSL reported a 100% ORR, with all patients demonstrating progression-free survival (PFS) and OS rates of 100% at 6 months (24). While numerous ongoing trials are investigating the combination of BTKIs with chemotherapy, molecular targeted drugs, and immune checkpoint inhibitors for first-line PCNSL treatment (**Table 1**), there are no large-scale, prospective, randomized controlled trials evaluating BTKIs as standalone or combinatorial therapies in newly diagnosed PCNSL. Additionally, no studies have examined the combination of BTKi with reduced-dose radiotherapy.

**Table 1:** Ongoing clinical trials investigating BTKIs in newly diagnosed PCNSL.

NCT Number	Phase	Conditions	Interventions	Treatment setting
NCT06646211	II	PCNSL	Zanubrutinib-Thiotepa-Methotrexate ± ASCT ± Zanubrutinib	Induction+Maintenance
NCT05896007	II	PCNSL	Zanubrutinib-Rituximab-Methotrexate/Temozolomide	Induction+Maintenance
NCT06445257	II	PCNSL	Zanubrutinib-Rituximab-Methotrexate-Temozolomide ± ASCT + Zanubrutinib	Induction+Maintenance
NCT04938297	II	Primary/Secondary CNS Lymphoma	Rituximab-Zanubrutinib-Lenalidomide+Zanubrutinib/Lenalidomide	Induction+Maintenance
NCT05600660	II	PCNSL	Orelabrutinib-Rituximab-Methotrexate± ASCT+ Orelabrutinib	Induction+Maintenanc
NCT06454266	II	PCNSL	Orelabrutinib-Rituximab-Methotrexate	Induction
NCT05036577	I	PCNSL	Orelabrutinib-Rituximab-Methotrexate-Dexamethasone+ Orelabrutinib	Induction+Maintenance
NCT05549284	II	PCNSL	Orelabrutinib-Rituximab-Methotrexate± ASCT/WBRT+ Orelabrutinib	Induction+Maintenance
NCT05390749	II	PCNSL	Orelabrutinib-Pomalidomide-Rituximab+ Methotrexate-Orelabrutinib-Rituximab	Induction
NCT05334238	III	PCNSL	ASCT± Orelabrutinib	Maintenance
NCT06901999	II	PCNSL	MTX/Thiotepa-Rituximab-Cyclophosphamide-Doxorubicin -Vincristine-Prednisone-Orelabrutinib± ASCT/WBRT+ Orelabrutinib	Induction+Maintenance
NCT04831658	I/II	PCNSL	Orelabrutinib-PD-1-Fotemustine	Induction
NCT04462328	I	Primary/Secondary CNS Lymphoma	Durvalumab-Acalabrutinib	Induction
NCT02203526	I	PCNSL	Temozolomide-Etoposide-Doxil-Dexamethasone-Ibrutinib-Rituximab	Induction
NCT04514393	II	PCNSL	Methotrexate-Ibrutinib-Temozolomide+ Ibrutinib	Induction+Maintenance
NCT02623010	II	PCNSL	Ibrutinib	Maintenance
NCT05211336	I	Primary/Secondary CNS Lymphoma	Venetoclax-Ibrutinib-Prednisone-Obinutuzumab-Lenalidomide	Induction
NCT04446962	I/II	PCNSL	Lenalidomide-Ibrutinib-Rituximab-Methotrexate -Procarbazine-Vincristin± ASCT	Induction
NCT05998642	II	PCNSL	Methotrexate-Rituximab-Ibrutinib+ Ibrutinib	Induction+Maintenance
NCT06541665	I	PCNSL	Tirabrutinib-Rituximab-Methotrexate-Procarbazine-Vincristine	Induction
NCT06940791	II	PCNSL	Tirabrutinib	Maintenance

PCNSL, Primary Central Nervous System Lymphoma; CNS, Central Nervous System; BTKIs, Bruton’s Tyrosine Kinase Inhibitors; ASCT, Autologous Stem Cell Transplantation.

WBRT is a standard treatment for newly diagnosed PCNSL, but it is associated with significant neurocognitive side effects, particularly when doses exceed 40 Gy—a concern for elderly patients (7). To mitigate the neurotoxic effects while preserving the therapeutic benefits of radiotherapy, studies have often employed reduced doses, such as 23.4 Gy in 13 fractions (8, 9). The RTOG 1114 trial, for example, compared R-MVP-A with 23.4 Gy WBRT to R-MVP-A alone, with preliminary results showing a significant improvement in 2-year PFS (78% vs. 54%). Guidelines now recommend reduced-dose WBRT as consolidation therapy for patients in complete remission (CR) following high-dose MTX induction (25). However, prospective data supporting rd-WBRT as a curative treatment are still lacking. A retrospective study by Song et al. found a median OS of 8 months for patients receiving WBRT, compared to 3.3 months for those receiving supportive care alone (P = 0.005). The study also showed no survival benefit from increasing the radiation dose to the whole brain (P = 0.10) (26). In another study of 66 chemotherapy-ineligible PCNSL patients, the 3-year OS was 74.9% in low-WBRT (30 Gy or less) and 72.8% in high-WBRT(more than 30 Gy) group without statistical difference (p = 0.533), although severe neurotoxicity was significantly more common in the high-dose group (27).

Given the limitations of radiotherapy alone, we combined rd-WBRT with orelabrutinib in elderly, chemotherapy-ineligible patients. All three patients were considered ineligible for standard HD-MTX–based chemotherapy due to advanced age, comorbidities, and baseline functional or cognitive status. We systematically assessed functional and cognitive capacity using ECOG performance status and MMSE scores; however, we acknowledge that these assessments do not fully capture the multidimensional health status of elderly patients, such as nutritional status, frailty, polypharmacy, and psychosocial support. Comprehensive or simplified geriatric assessment tools could provide a more objective evaluation of fitness for intensive therapy, which is a limitation of the current study. This combined approach aims to achieve rapid local disease control via rd-WBRT while simultaneously providing systemic disease management through BTKI therapy.

Regarding safety, no radiotherapy-induced neurotoxicity was observed in our cohort of three patients, including symptoms such as progressive severe cognitive dysfunction, ataxia, or urinary incontinence. Adverse events typically associated with BTKIs are related to immunosuppression and hematologic toxicity, including neutropenia, lymphopenia, anemia, thrombocytopenia, and opportunistic infections, such as aspergillosis and Pneumocystis jirovecii pneumonia (PCP) (28). However, none of these complications occurred in our patients. Moreover, no significant non-hematologic adverse events occurred, including cardiovascular events, hepatic or renal toxicity, or other organ dysfunction. Notably, one patient had a prior history of cerebral infarction and was receiving long-term indobufen therapy. To mitigate the risk of bleeding, indobufen was discontinued, and orelabrutinib was administered to manage the cerebral infarction. Importantly, no recurrence of cerebral infarction was observed during the 2-year follow-up period.

This case series highlights the potential utility of combining reduced-dose whole brain radiotherapy with orelabrutinib in elderly PCNSL patients who are unfit for standard chemotherapy. All patients initially achieved meaningful radiologic and clinical responses, and two maintained durable remission. One patient experienced disease relapse only after discontinuing orelabrutinib and switching to an alternative regimen. The combination regimen was well tolerated, with no significant neurotoxicity or hematologic complications observed. These findings support further investigation of BTKi-radiotherapy combinations as a potential treatment strategy for vulnerable populations with PCNSL.

## Data Availability

The original contributions presented in the study are included in the article/supplementary material. Further inquiries can be directed to the corresponding author.
